# The effect of daily life activities on intraocular pressure related variations in open-angle glaucoma

**DOI:** 10.1038/s41598-021-85980-2

**Published:** 2021-03-23

**Authors:** Kevin Gillmann, Robert N. Weinreb, Kaweh Mansouri

**Affiliations:** 1 Glaucoma Research Center, Montchoisi Clinic, Swiss Visio, Lausanne, Switzerland; 2 Hamilton Glaucoma Center, Shiley Eye Institute and Viterbi Family Department of Ophthalmology, University of California San Diego, La Jolla, CA USA; 3 Department of Ophthalmology, University of Colorado School of Medicine, Denver, CO USA; 4 Glaucoma Service, Moorfields Eye Hospital NHS Foundation Trust, London, UK

**Keywords:** Risk factors, Ocular hypertension

## Abstract

The recent advent of continuous intraocular pressure (IOP) telemetry has led to an increased awareness of the importance of IOP fluctuations, and theories have emerged that IOP variations could play as much a role in glaucoma progression as the mean level of IOP. The aim of the present study was to evaluate the direct effect of common daily activities on IOP-related profiles. Primary open-angle glaucoma and glaucoma suspect patients were prospectively enrolled from specialist clinics at the University of California San Diego (UCSD), USA. Patients were fitted with a SENSIMED Triggerfish (TF) contact lens sensor (CLS) and were instructed to return to their usual daily activities for 24 h. They were asked to record each specific activity or event in a diary. The protocol was repeated twice. The following events were recorded: “walking/cycling”, “resistance training”, “yoga/meditation”, and “emotional stress”. CLS measurements recorded 60-to-30 min prior to each event were used as a baseline reference, and all IOP-related fluctuations for 120 min after the start of each event were reported in relation to this reference. Forty relevant events from 22 CLS recordings in 14 patients were retrieved from the diaries. Walking/cycling (n = 10) caused a small but statistically significant elevation of the IOP-related profile during the activity (*p* = 0.018). Resistance training (n = 11) caused a persistent elevation of the IOP-related profile from the onset of the activity (*p* = 0.005) through 120 min after the activity was stopped (*p* = 0.007). Yoga/meditation (n = 4) caused a sustained drop in the IOP-related profiles through to 120 min, although this was not statistically significant (*p* > 0.380). Emotional stress (n = 13) was associated with a gradual elevation of the IOP-related profile from the start of the stressful stimulus. Both early and late variations were statistically significant (*p* = 0.038 and *p* = 0.021, respectively). The present study suggests that emotional stress and resistance training may be associated with persistent IOP-related profile elevation.

## Introduction

Although glaucoma is the leading cause of irreversible blindness worldwide, the exact pathophysiology and biological mechanisms underlying the disease are still poorly understood^1^. It has been clearly documented that intraocular pressure (IOP) plays a critical role in the disease process and that elevated IOP is one of the main risk factors for glaucoma progression^2,3^. Yet, in recent years, the advent of continuous IOP telemetry has led to an increased awareness of the importance of IOP fluctuations, and theories have emerged that IOP variations could play as much a role in glaucoma progression as the mean level of IOP^4–6^.

The SENSIMED Triggerfish (TF) contact lens sensor (CLS) (Sensimed, Lausanne, Switzerland) was the first commercially available device to permit continuous monitoring of IOP-related variations over 24 h. The device consists of a silicone soft contact lens within which a miniaturized telemetric sensor detects subtle fluctuations in ocular volume^7^. While changes in ocular volume were associated with IOP variations, TF CLS do not provide actual IOP measurements, but rather a representation its dynamic variations. Yet, its accuracy and sensitivity in measuring IOP variations was confirmed in several studies^8–10^.

A number of lifestyle choices and daily activities were shown to have a direct effect on IOP. Namely, resistance training^11,12^, frequent alcohol consumption^13^, psychological stress^14,15^ and head-down^16^ or supine body positions^17^ were suspected to increase IOP, while meditation^18^, sexual activity and orgasm^19^ were suspected to cause IOP reduction. Yet, most of these studies are either based on population surveys or laboratory experiments and base their observations on a small number of measurements using rebound tonometry. At present, data on the real-time effect of these activities in real life are mostly anecdotal.

The aim of the present study was to evaluate the direct effect of common non-standardized daily activities on IOP-related profiles of a cohort of open-angle glaucoma patients.

## Methods

### Study design

This was a prospective single-centre study, conducted at the University of California San Diego (UCSD), USA. The study was approved by the University of California San Diego Institutional Review Board (IRB) and conducted in full compliance with the Declaration of Helsinki. Written informed consent was obtained from all patients.

### Subject inclusion

Patients were recruited from a specialist glaucoma clinic, and every effort was made to enrol all suitable patients who attended the clinic. Patients were enrolled in the study if they met the following criteria: open-angle glaucoma or suspect, age between 18 and 80 years, best-corrected visual acuity of ≥ 20/200 in the study eye, astigmatism ≤ 2.00 D. Individuals with any of the following were excluded: any history of intraocular surgery, severe dry eyes syndrome, corneal abnormalities, contraindications for contact lens wear, known intolerance to silicone, pregnancy or lactation. Glaucoma was defined as the association of repeatable visual fields defects (persistent scotoma on at least two consecutive standard automated perimetry tests [Octopus, Haag Streit, Koeniz, Switzerland] with a test reliability index ≥ 15%), and an abnormal optic disc appearance (presence of neuroretinal rim thinning or localized or diffuse retinal nerve fiber layer [RNFL] defects) indicative of glaucoma as observed under slit-lamp examination or on SD-OCT imaging (Spectralis OCT, Heidelberg Engineering AG, Germany). Glaucoma suspects were defined as having an abnormal optic disc appearance and/or persistent ocular hypertension with normal visual fields. Gonioscopy was performed on all eyes prior to enrolment to ensure no closed angles were included^20^.

### Instrumentation

The SENSIMED Triggerfish CLS consists of an oxygen-permeable soft contact lens fitted with two resistive strain gauges that are capable of recording ocular dimensional changes in the area of the corneo-scleral junction the outcome of which have been shown to be related to IOP^17,21^. The disposable contact lens exists in 3 different base curves, chosen based on keratometry values. The output of the sensor is expressed in millivolt (mV) equivalents. The CLS device records consecutive samples at 10 Hz during 30 s every 5 min throughout the recording period (up to 288 30-s data sets over 24 h). The medians of each 30-s data set were used to build individual CLS output curves. The device has been described in more detail elsewhere^6^.

### Measurement protocol

After inclusion, central corneal thickness (CCT) and keratometry were measured for all eyes using a Pentacam camera (Oculus, GmbH). Intraocular pressure was then measured by one investigator using a Goldmann applanation tonometer (GAT) under topical oxybuprocaine^22^. Two independent measurements were made in each eye and the mean value was reported. Individual keratometries were used to choose the most appropriate contact lens base curve and patients had a SENSIMED Triggerfish CLS fitted. The eye in which the CLS was fitted was chosen at random^23^. Patients were instructed to return to their usual daily activities, and to record the start and end time of each specific activity or event in a diary. Full-frame metal glasses were prohibited throughout the monitoring since they are known to interfere with the device energy and data transfer. Twenty-four hours later, the patients returned to the investigation centre to have the CLS removed. Intraocular pressure was then measured again using the same measurement protocol as the day before. The protocol was repeated twice for each patient in the same eye, at least 7 days apart.

### Data treatment

Recorded events were divided into 5 groups based on the descriptions provided by subjects: “walking and cycling”, “resistance training”, “yoga and meditation”, “emotional stress” and “alcohol consumption”. Events not fitting any of these definitions were disregarded. For each event, all CLS measurements recorded within the following timeframes were extracted and averaged: 60-to-30 min prior to the event, during the event, 0-to-30 min after the event, 30-to-60 min after the event, and 90-to-120 min after the event.

### Statistical analysis

Recordings were considered low quality and were excluded from the analysis when less than 80% of all 24-h measurements were valid. The average of all CLS measurements recorded 60-to-30 min prior to each event were used as a baseline reference (0 mVeq), and all subsequent IOP-related fluctuations were reported in relation to this reference. To assess the effect of each type of event on IOP-related profiles, individual variations at each timepoint were compared to the reference value using a paired t-test. *P* values < 0.05 were considered statistically significant.

## Results

### Cohort

Forty-one eyes of 41 patients were enrolled, and underwent a total of 82 CLS recordings. All recordings were considered of good quality. In total, 40 relevant events from 22 CLS recordings in 14 patients were retrieved from the subjects’ diaries (n = 10 [Walking and cycling]; n = 11 [Resistance training]; n = 4 [Yoga and meditation]; n = 13 [Emotional stress]; n = 2 [Alcohol consumption]). Table 1 presents the patients’ demographics and characteristics.

**Table 1:** Demographics and characteristics of enrolled patients who reported relevant events in their diaries (POAG: Primary open-angle glaucoma; MD: Mean defect; PSD: Pattern standard deviation; IOP: Intraocular pressure; CCT: Central corneal thickness).

Demographic	n = 14
Right/left	6 (42.9%)/8 (57.1%)
Male/female	8 (57.1%)/6 (42.9%)
White/African/Asian	12 (85.7%)/1 (7.1%)/1 (7.1%)
Age (years)	57.9 ± 13.0
POAG/suspect	8 (57.1%)/6 (42.9%)
Visual field MD (dBs)	− 1.3 ± 2.7
Visual field PSD (dBs)	2.8 ± 1.9
Pre-fitting IOP (mmHg)	17.7 ± 3.9
CCT (μm)	557.9 ± 39.6
Smokers	1 (7.1%)
Weight (kg)	78.4 ± 17.3
Height (cm)	171.5 ± 10.5

### Observed effects

**Figure 1:**
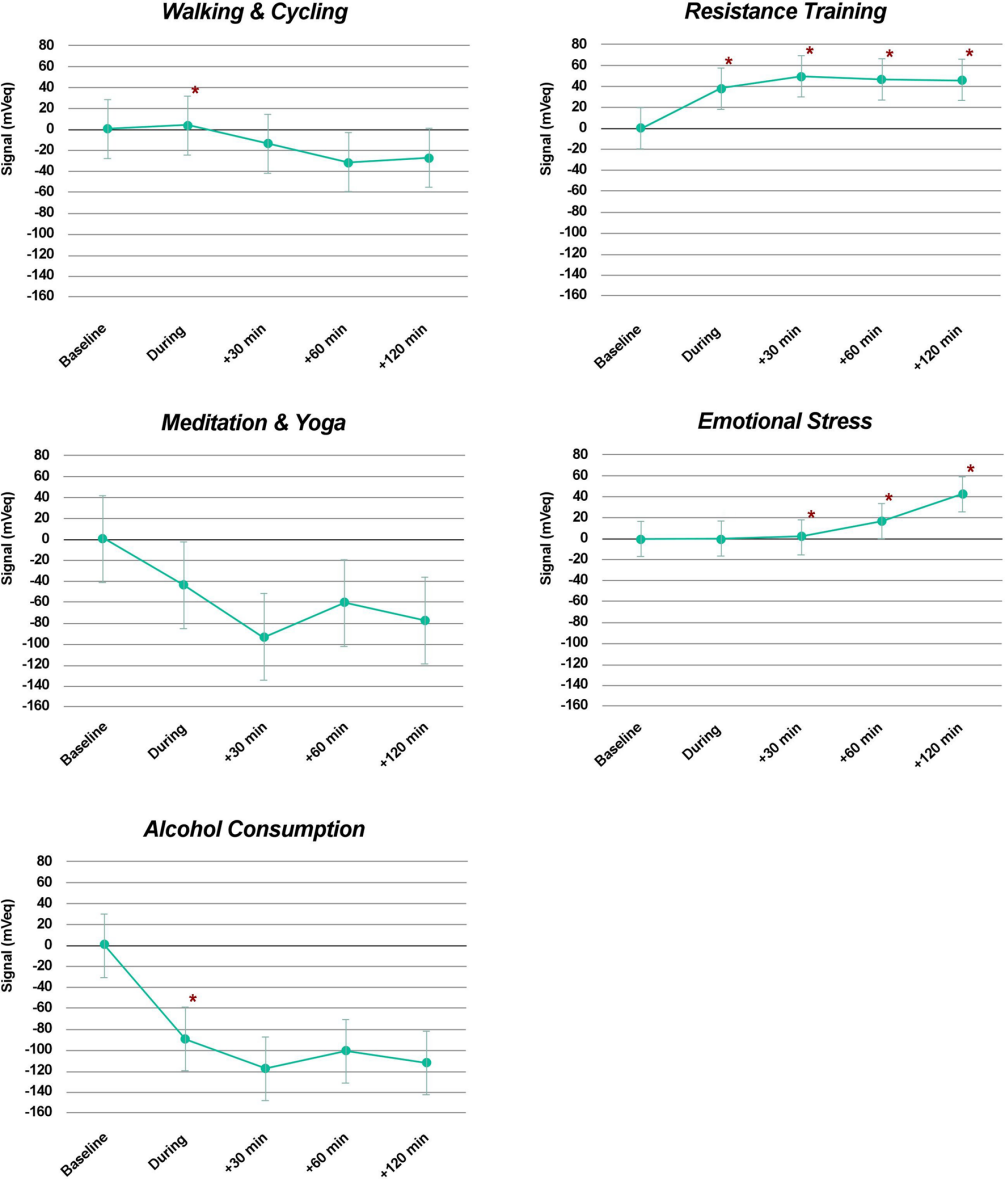
Mean effect of each group of activities on intraocular pressure-related signal recorded in mVeq, during the event, within 30 min of the end of the event, between 30 and 60 min of the end of the event, and between 90 and 120 min of the end of the event, compared to baseline measurements set at 0 mVeq (30–60 min before the start of the event). Vertical bars represent the 95% confidence interval, and asterisks represent statistically significant differences from baseline (*p* < 0.05).

On average, “walking and cycling” caused a small but statistically significant elevation of the IOP-related profile during the activity (*p* = 0.018), followed by a non-significant signal decrease. “Resistance training” caused a persistent elevation of the IOP-related profile from the onset of the activity (*p* = 0.005), that remained statistically significant through 120 min after the activity was stopped (*p* = 0.007). “Yoga and meditation” caused a sustained drop in the IOP-related profiles through to 120 min, but none of the associated variations were statistically significant (*p* > 0.380). “Emotional stress” was associated with a gradual elevation of the IOP-related profile from the start of the stressful stimulus. Both early and late variations were statistically significant (*p* = 0.038 and *p* = 0.021, respectively). Alcohol was associated with a statistically significant reduction of the IOP-related profile at the time of consumption (*p* = 0.049), but subsequent variations were not statistically significant. Figure 1 shows the average variation in IOP-related profiles following each of the analyzed event.

## Discussions

The present study confirms the effect of physical activities, emotional state and alcohol consumption on IOP-related profiles.

The reported results confirm the association between resistance training and IOP elevation that had been previously described by Vera et al.^24^. In a study of 25 healthy volunteers, they identified an instant IOP elevation during resistance training that was directly correlated to the intensity of the effort and to the size of the muscle trained^25^. Interestingly, they described a prompt reduction of IOP to baseline within 10 s after the end of muscle contraction, while our measurements suggest prolonged elevation through to 120 min. This may be explained by differences in methods and measurement techniques. Indeed, while Vera et al. used single groups of 6 rapid consecutive rebound tonometer readings at the start, during and at the end of the exercise period to evaluate its effect, the automated nature of the CLS recording did not permit the matching of recording bursts with specific phases of the exercise. Therefore, we have averaged all the CLS recordings over 30-min-long periods before and for up to 120 min after resistance training to evaluate any lasting effect. While not movement-synchronized, the 1800 samples recorded during every 30-min period are expected to provide a more faithful representation of the short-term IOP-related fluctuations following each event. Furthermore, the IOP-related profile elevation within the 30 min following resistance training may simply represent the effect of short-term effort-related hormone release. Finally, differences in populations may explain some of the differences observed, since Vera et al. had enrolled healthy young adults, as compared to a population with, on average, older adults with a diagnosis of glaucoma or glaucoma suspect in the present study. Further research will be needed to explore the short- and long-term effects of resistance training on IOP.

To the best of our knowledge, the effect of walking or cycling on IOP had not been previously studied. The increase in IOP-related profile during “walking or cycling” was an unexpected finding since prolonged erect position was associated with gravitational IOP reduction^26^, and endurance training was previously associated with the systemic release of dopamine^27^, that was subsequently shown to down-regulate the production of aqueous humour through the release of norepinephrine triggered by the activation of DA2 and DA3 receptors^28^. Yet, several theories may explain this finding: (1) DA1 receptors found in the ciliary body were shown to have the opposite effect to DA2 and DA3 receptors, and up-regulate the production of aqueous humour. Yet, to date, it is unclear how endogenous serotonin influences aqueous production. (2) Endurance training was associated with wide variations in plasma levels of a number of other neurotransmitters and hormones that may have different effects on the control of IOP. Amongst them, serotonin was shown to be significantly reduced by regular training^29^, and cross-sectional studies of patients using serotonin reuptake inhibitors have suggested an association between lower levels of serotonin and higher IOP^30^. (3) Since the intensity of the activity was not recorded, intense Walking or cycling, either uphill or for extended periods of time, might have had the same physiological effect as resistance training. (4) Participants were not specifically instructed to monitor their fluid intake, which may have a cofounding effect. (5) In a relatively small cohort, individual variations, whether they be in terms of ocular physiology, hydration, environment, effort intensity or recovery, may have affected the statistical significance of late IOP-related profiles. These reasons might explain the conflicting reports on the effect of regular exercise in the literature^31–35^.

The present study showed a non-significant drop in IOP-related profile during and after Meditation and yoga. While Jasien et al. demonstrated a marked increase in IOP in all head-down yoga positions evaluated^36^, large studies concentrating on the effect of meditation defined as simple mindfulness reported a significant persistent IOP-lowering effect associated with the daily practice of the activity for 21 consecutive days^37^. Thus, the non-significance of our results may be explained (1) by the inclusion of both yoga and meditation within the same analysis, potentially confusing the results depending on the yoga positions practiced by the subjects, (2) by the occasional practice of meditation in the studied subjects, suggesting the importance of daily practice to achieve persistent effect, and (3) by the relatively small number of Meditation and yoga sessions recorded in the present study.

Over the years, a number of conflicting reports were published on emotional stress, and its effect on IOP remains debated^38^. While Khezri et al. did not observe any significant association between the pharmacological reduction of anxiety and IOP^39^, several authors reported an increase in IOP during standardized stressful events. Indeed, in a study in which volunteers were asked to perform stressful mental arithmetic tasks, Brody et al. showed that these caused a mean increase in IOP of 1.3 mmHg^40^. In another study, Méndez-Ulrich et al. observed that self-reported anxious study subjects had a significantly higher IOP than calm volunteers^41^. Several factors may account for these seemingly contradicting results. First, it is evident that emotional stress is a subjective experience by nature, and the perception of an event may vary widely between subjects^42^. Therefore, while it may be possible to standardise a stressful stimulus, it is impossible to replicate the subjective experience of stress perceived by the subjects. Then, several other factors were shown to influence individual reactions to stress and IOP variations, including age and personality types. For instance, Bubella et al. showed that type A personalities exhibited greater IOP variability than other types of personality^43^. The methodology of the present study may contribute to the statistical relevance of its results over standardized protocols or pharmaceutical intervention. Indeed, the fact that the stressful stimuli were self-reported real-life events allowed subject to only report events which they subjectively perceived as stressful, based on their own personality and emotional threshold.

Alcohol consumption is often considered detrimental to glaucoma patients. Yet, reports on the direct effect of dietary ethanol on IOP are rare, and the reported association between alcohol consumption and glaucoma progression may owe, in part, to its neurotoxic effect^44,45^. A single case study describes a systematic transient reduction in IOP following the consumption of Champagne wine in a 66-year-old glaucoma patient^46^. This may potentially be explained by the biphasic effect of alcohol, initially causing a decrease in blood pressure for up to 12 h, followed by a subsequent increase^47^, which may be mirrored by IOP as suggested by Takashi et al. in a large cross-sectional study of 1569 subjects showing a positive association between systolic blood pressure and IOP^48^. The present results are in keeping with this observation, yet the very small number of observations, both from the same subject, certainly affect their significance and generalisation.

The above observations highlight the significant influence of most common daily activities on IOP, which may be especially relevant to glaucoma patients. This supports the importance of lifestyle counselling in patients with glaucoma. Furthermore, this may support the use of telemetry for 24-h IOP variability monitoring in borderline glaucoma cases to help clinicians weigh up the potential benefits of early surgical intervention based on patients’ “normal” daily activities^49–51^. Yet, the effect of surgical treatments on out-of-office IOP fluctuations would need to be ascertained.

### Study limitations

This study has several limitations. First, its cohort was relatively small with only 41 eyes and even fewer patients partaking in each activity. Thus, while some statistically significant effect was observed, the very small numbers of “alcohol consumption” and “yoga and meditation” events reduce the value of these findings, and the authors suggest these may only be viewed as anecdotal observations. Second, the real-life nature of this study relied on non-standardized self-reported activities. While this induces the inherent bias of not controlling for intrinsic and extrinsic parameters such as temperature, heart rate, dehydration, fluid intake, body or head position, activity intensity or potential simultaneous activities, the aim of the study was to take advantage of the out-of-office continuous recording allowed by the CLS to study the effect of day-to-day events on IOP with as little interference as possible. Furthermore, it was decided to compare the average measurements obtained over 30-min recording periods (1800 samples) to minimize the biases associated with sporadic events or approximative self-reported timings. Thus, while caution may be used before generalizing the present results, this study constitutes, as far as we are aware, the first observation of the effect of a range of real-life, non-standardized, daily activities on IOP-related profiles using 24-h continuous CLS monitoring. Further real-life studies based on larger cohorts to explore the effect of more day-to-day events are warranted, and will be permitted by the generalization of IOP telemetry technology. These should to be complemented with more standardized studies to confirm the specific factors influencing the observed variations.

## Conclusions

The present study suggests that emotional stress and resistance training may be associated with persistent IOP-related profile elevation. More research is warranted to confirm these findings and assess the effect of glaucoma surgery on IOP fluctuations associated with day-to-day activities.
